# Lightweight SNR-Adaptive Receiver-Side Enhancement for DeepJSCC-Based Wireless Image Transmission

**DOI:** 10.3390/s26165134

**Published:** 2026-08-14

**Authors:** Shouquan Hou, Peng Zhao, Nuo Chen

**Affiliations:** College of Operational Support, Rocket Force University of Engineering, Xi’an 710025, China; 17334402125@163.com (S.H.);

**Keywords:** semantic communication, DeepJSCC, perceptual enhancement, SNR-adaptive, frequency recovery, lightweight neural networks, wireless image transmission

## Abstract

Deep joint source-channel coding (DeepJSCC) has emerged as a promising paradigm for semantic-aware wireless image transmission, achieving strong performance under challenging channel conditions. However, MSE-trained DeepJSCC systems typically achieve high peak signal-to-noise ratio (PSNR) values but suppress high-frequency details, resulting in perceptually blurry reconstructions that fail to capture fine textures and edge information. Existing perceptual enhancement approaches for JSCC systems face significant practical limitations: full transceiver redesign methods require replacing both the transmitter and the receiver with large models (19–31 million parameters), incurring substantial deployment costs; diffusion-based refinement approaches require over 1700 million additional parameters and introduce inference latency exceeding 13 s, rendering them unsuitable for latency-constrained wireless applications; and generic image restoration networks lack channel state awareness and cannot adapt to varying signal-to-noise ratio (SNR) conditions. This paper proposes a lightweight receiver-only perceptual enhancer designed for use with frozen DeepJSCC backbones. The proposed module adopts residual learning with feature-wise linear modulation (FiLM)-based SNR-adaptive modulation to dynamically adjust the enhancement strength under varying channel conditions. A radially weighted FFT magnitude loss is further introduced to guide high-frequency recovery. The enhancer adds only 0.29 million trainable parameters (<1% of the backbone) and requires neither transmitter modification nor backbone retraining. Extensive experiments on the Kodak24 and DIV2K datasets demonstrate a 34.4–37.5% LPIPS reduction over the frozen DeepJSCC baseline under AWGN channels. Supplementary robustness evaluations further show a 30–33% LPIPS reduction under Rayleigh fading, and stable generalization to unseen SNR levels. The receiver-side decoder-plus-enhancer pipeline requires 43 ms at 768 × 512 resolution, corresponding to approximately 23 frames per second.

## 1. Introduction

The rapid evolution of wireless communication systems toward sixth-generation (6G) networks has accelerated a paradigm shift from conventional bit-oriented transmission to semantic-aware communication [1,2,3,4]. This transition is driven by the exponential growth of multimedia traffic and the increasing demand for intelligent services that prioritize the efficient delivery of semantic information rather than the exact reconstruction of every transmitted bit [2,5,6]. Deep joint source-channel coding (DeepJSCC) has emerged as a key enabling technology within this paradigm, allowing image transmission over wireless channels to be optimized end-to-end by jointly learning source compression and channel coding in a unified neural network framework [7,8,9].

Since the pioneering work of Bourtsoulatze et al. [7], DeepJSCC has received considerable research attention because of its ability to adapt to channel conditions and outperform conventional separate source-channel coding schemes, particularly under bandwidth-limited and low-SNR regimes [10,11,12]. Subsequent studies have incorporated attention mechanisms [13,14] and transformer architectures [15] to improve feature extraction and channel adaptation. Nevertheless, a fundamental challenge remains: DeepJSCC models trained with mean squared error (MSE) loss inherently prioritize distortion minimization, which, as theoretically established by Blau and Michaeli [16], inevitably suppresses high-frequency image content. As a result, such models often produce reconstructions with high peak signal-to-noise ratio (PSNR) but limited perceptual quality, manifested as over-smoothed textures and the loss of fine details [17].

The perception–distortion tradeoff [16] has motivated the development of perceptually oriented image transmission methods. In the broader image restoration literature, generative adversarial networks (GANs) [18,19,20] and perceptual losses [21,22,23] have been shown to effectively recover high-frequency details. Recent studies have extended these ideas to JSCC systems. For example, SwinJSCC [15] incorporates Swin transformer blocks to improve feature representation, whereas PLSCC [24] explicitly optimizes perceptual quality through adversarial training. However, these methods require a complete transceiver redesign with models containing 19–31 million parameters, thereby imposing substantial deployment costs on existing systems.

More recently, diffusion models have demonstrated remarkable capability in high-fidelity image synthesis and restoration [25,26,27,28]. DiffJSCC [29] applies diffusion-based refinement at the receiver and achieves strong perceptual quality. However, this approach requires more than 1700 million additional parameters and incurs an inference latency exceeding 13 s for a single image, making it unsuitable for real-time wireless applications with stringent latency constraints. Generic image restoration networks, such as DnCNN [30], NAFNet [31], and SwinIR [32], are more computationally efficient, but they are not aware of channel states and therefore cannot adapt the enhancement intensity to varying signal-to-noise ratio (SNR) conditions [33].

These existing approaches can be broadly categorized by their deployment requirements and channel awareness: (i) full transceiver redesign methods (SwinJSCC, PLSCC) require replacing both the transmitter and the receiver with large models (19–31 M parameters), incurring substantial infrastructure costs; (ii) receiver-side diffusion refinement (DiffJSCC) preserves the transmitter but demands over 1700 M additional parameters and iterative sampling, making it impractical for latency-constrained deployment; and (iii) generic post-processing networks (DnCNN, NAFNet, SwinIR) are lightweight and receiver-only but operate without any knowledge of channel conditions, applying a fixed transformation regardless of SNR. The proposed enhancer occupies a distinct design point that combines the strengths of these categories while avoiding their limitations: it is receiver-only and lightweight (0.29 M parameters, <1% of the backbone) like generic post-processing, yet channel-aware through FiLM-based SNR conditioning like full transceiver redesign—without requiring transmitter modification, backbone retraining, or diffusion-scale computation.

This paper addresses the practical gap between deployment feasibility and perceptual quality enhancement for existing DeepJSCC systems. We propose a lightweight receiver-only perceptual enhancer designed specifically for frozen DeepJSCC backbones, enabling perceptual improvement without modifying deployed transmitters or retraining existing infrastructure. The key insight is that a carefully designed lightweight module can recover high-frequency information suppressed by MSE training while adapting to channel conditions through SNR-conditioned modulation. The main contributions of this work are summarized as follows:

1. We propose the first receiver-only plug-and-play perceptual enhancer for deployed DeepJSCC systems. The proposed approach maintains compatibility with existing infrastructure by keeping both the encoder and decoder frozen, while introducing only 0.29 million trainable parameters (<1% of the backbone).
2. We design an SNR-adaptive residual enhancement mechanism based on FiLM modulation [34], enabling a single lightweight model to dynamically adjust restoration strength according to channel quality. Under low-SNR conditions, the mechanism applies conservative enhancement to reduce the risk of noise amplification, whereas under high-SNR conditions it enables stronger high-frequency recovery.
3. We introduce a radially weighted FFT magnitude loss that provides spectral-domain supervision complementary to spatial perceptual losses. This frequency-targeted guidance directly addresses the high-frequency suppression commonly observed in MSE-trained DeepJSCC models.
4. We conduct comprehensive experiments on the Kodak24 and DIV2K datasets under AWGN channels, demonstrating consistent LPIPS improvements with 34.4–37.5% relative reduction, while incurring minimal computational overhead, including 29.8 ms enhancer-only latency, 216 GFLOPs, and modest receiver-side deployment cost.

## 2. Related Works

### 2.1. Deep Learning for Joint Source-Channel Coding

The concept of joint source-channel coding can be traced back to the foundations of information theory [1]; however, practical implementations remained elusive until the advent of deep learning [10]. Bourtsoulatze et al. [7] pioneered DeepJSCC for wireless image transmission, demonstrating that neural networks can jointly learn to compress image data and protect it against channel noise. Their encoder–decoder architecture, trained end-to-end with channel noise augmentation, outperformed conventional separate coding schemes, particularly in bandwidth-limited and low-SNR regimes [17,35].

Subsequent studies have enhanced DeepJSCC through attention mechanisms and transformer architectures [36]. Xu et al. [13] introduced spatial and channel attention modules to improve feature selection and channel adaptation. Yang [14] proposed WITT, which employs transformer-based architectures for improved global context modeling. More recently, SwinJSCC [15] leveraged Swin Transformer blocks to achieve state-of-the-art performance and improved perceptual quality through more effective feature extraction.

Constellation-constrained variants have also emerged. DeepJSCC-Q [37] incorporates discrete constellation constraints, thereby bridging the gap between learned representations and practical modulation schemes. Extensions to video transmission [38] and transformer-based semantic communication [39] have further broadened the applicability of deep JSCC. Task-oriented semantic communication frameworks [40,41] optimize transmission for specific downstream tasks. Together, these advances have established DeepJSCC as a promising technology for semantic communication in 6G networks [2].

### 2.2. Perceptual Quality Optimization in Image Transmission

The fundamental tradeoff between distortion and perception was formally characterized by Blau and Michaeli [16], who showed that minimizing distortion, such as MSE, inevitably compromises perceptual quality. This insight has important implications for image transmission: PSNR-oriented reconstructions tend to appear blurry because high-frequency details, which contribute only marginally to MSE, are often suppressed.

In the image restoration domain, GAN-based methods [18,19] and perceptual losses [21,22] have been widely adopted to alleviate this problem. Deep feature consistency [42] and attention-based restoration networks [43] have further improved perceptual quality. LPIPS [21], which measures perceptual similarity using deep network features, has become a standard metric for perceptual quality assessment [23]. For JSCC systems, PLSCC [24] incorporated adversarial training to improve perceptual quality, achieving significant LPIPS gains at the cost of a complete transceiver redesign.

Diffusion models [25] represent a recent advance in generative restoration. Transformer-based restoration architectures [44,45,46] have achieved favorable efficiency–quality tradeoffs. DiffJSCC [29] applies conditional diffusion at the receiver side and achieves remarkable perceptual fidelity. Denoising diffusion restoration models [47] and image super-resolution via iterative refinement [27] have demonstrated state-of-the-art performance. Conditioning methods for diffusion models [48,49] enable fine-grained control over the generation process. Generative semantic communication [50] and reliable semantic transmission frameworks [51] have explored related directions. However, the computational cost of diffusion-based approaches—exceeding 1700 million parameters and requiring approximately 13 s of inference time—limits their practical deployment in wireless scenarios with stringent latency constraints.

The above methods can be organized along two axes—deployment scope and channel awareness—to clarify their relationship to the present work. Full transceiver redesign methods (PLSCC, SwinJSCC) achieve strong perceptual quality but require retraining both the encoder and decoder, making them impractical for already-deployed systems. Receiver-side diffusion refinement (DiffJSCC) avoids transmitter modification but introduces prohibitive computational overhead. Generic image restoration networks (DnCNN, NAFNet, SwinIR) are efficient and receiver-only, yet they lack channel-state information and apply SNR-agnostic processing. The proposed approach differs from all three categories by combining the receiver-only, lightweight properties of generic restoration with the SNR-adaptive behavior of channel-aware methods, while requiring neither transmitter modification nor backbone retraining.

### 2.3. Adaptive Neural Networks for Wireless Communications

Adaptation to channel conditions is essential for wireless systems. FiLM [34] provides a general mechanism for conditioning neural networks on external information through feature-wise affine transformations. Although originally proposed for visual reasoning, FiLM has been applied to a variety of adaptive systems [52,53]. Feature pyramid networks [54] enable multi-scale feature processing and enhance representation learning.

Channel-aware neural networks for communications often incorporate SNR information through various conditioning schemes [55]. Attention-based mechanisms [56] enable dynamic feature recalibration. In this work, we leverage FiLM for SNR-adaptive modulation, allowing the enhancer to adjust its behavior according to channel quality—applying stronger enhancement under favorable channel conditions while adopting more conservative processing when noise levels are high.

**Novelty of FiLM usage.** While FiLM has been applied in visual reasoning [34] and feature-level conditioning [52], its use in receiver-side JSCC enhancement differs in three aspects. First, prior SNR-aware conditioning in JSCC systems (e.g., attention-based channel adaptation in SwinJSCC [15]) is applied within the end-to-end transceiver during joint training; in contrast, our FiLM modulation conditions a post-hoc enhancer that operates on the output of a frozen backbone, without access to intermediate encoder features. Second, we constrain the FiLM scale via a sigmoid function $\sigma \left(\gamma_{i}\right)\in \left(0,1\right)$, which provides a bounded energy gate specifically designed to prevent noise amplification at low SNR—a consideration absent in standard FiLM. Third, unlike generic image restoration networks that apply a fixed transformation regardless of channel state, our FiLM-based design is the first to integrate channel-conditioned modulation into a lightweight receiver-only post-processor for DeepJSCC.

## 3. Methods

### 3.1. System Model

**Figure 1:**
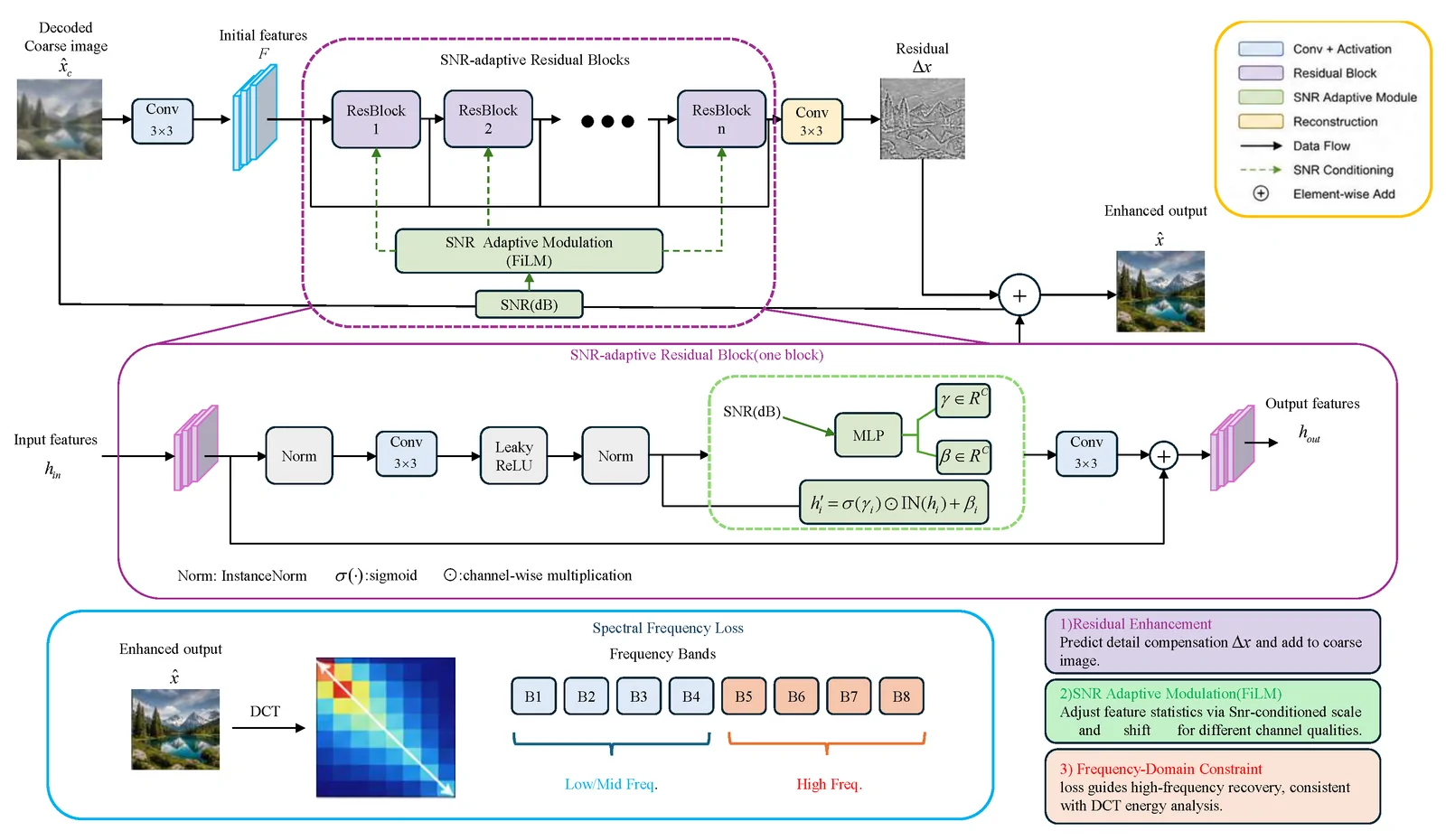
Overview of the proposed SNR-adaptive residual enhancement framework with FiLM-based modulation and frequency-domain constraint. Blue blocks denote the frozen DeepJSCC backbone; orange blocks denote the proposed receiver-side enhancer; green blocks denote the FiLM SNR-conditioning pathway. Arrows indicate the direction of data flow.

As illustrated in Figure 1, the proposed system augments a frozen DeepJSCC encoder–decoder backbone with a lightweight SNR-adaptive residual enhancer at the receiver side. Given an input image $x\in R^{H\times W\times 3}$, the frozen encoder $f_{E}\left(\cdot ;\theta \right)$ maps x into channel symbols, which are transmitted over an AWGN channel and then decoded by the frozen decoder $f_{D}\left(\cdot ;\psi \right)$ to obtain a coarse reconstruction:

(1) $${\hat{x}}_{c}=f_{D}\left(f_{E}\left(x;\theta \right)+n;\psi \right),$$

where $n∼N(0,\sigma^{2}I)$ denotes AWGN with variance $\sigma^{2}=10^{-\mathrm{SNR}/10}$. The channel bandwidth ratio (CBR) is defined as ρ=k/(3HW), where *k* is the number of transmitted channel symbols.

The DeepJSCC backbone is trained with the MSE loss $L_{\mathrm{MSE}}={∥x-{\hat{x}}_{c}∥}_{2}^{2}$, which typically yields high PSNR but limited perceptual quality. In particular, MSE-oriented optimization tends to preserve low-frequency structures while suppressing high-frequency details. To quantify this effect, let F(·) denote the two-dimensional discrete cosine transform (DCT). We define the energy retention rate of frequency band *b* as

(2) $$R\left(b\right)=\frac{∥F\left({\hat{x}}_{c}\right)\left[b\right]{∥}_{2}^{2}}{{∥F\left(x\right)\left[b\right]∥}_{2}^{2}}.$$

As analyzed in Section 4, high-frequency bands exhibit R(b)<0.1%, confirming severe high-frequency attenuation in the coarse DeepJSCC reconstruction.

**Problem Statement.** Given a deployed JSCC system with a frozen encoder–decoder pair $\left(f_{E},f_{D}\right)$, we aim to learn a lightweight receiver-side enhancer $f_{R}\left(\cdot ;\phi \right)$ that predicts a residual detail compensation term conditioned on both the coarse reconstruction and the channel SNR:

(3) $$\Delta x=f_{R}\left({\hat{x}}_{c},\mathrm{SNR};\phi \right),$$

and produces the enhanced image as

(4) $$\hat{x}={\hat{x}}_{c}+\Delta x.$$

Here, |ϕ|≪|θ|+|ψ|, and the backbone parameters θ and ψ remain frozen throughout both training and inference. This design requires no modification to the transmitter or the deployed DeepJSCC backbone, thereby preserving backward compatibility.

We assume that the receiver has access to the channel SNR, which serves as a conditioning input to the enhancer $f_{R}$. In practical systems, this value can be obtained through pilot-based channel estimation or received signal strength indicators. The robustness of the proposed enhancer to SNR estimation errors—including moderate mismatches that may arise from quantized feedback or slow channel estimation—is analyzed in Section 5.

### 3.2. Network Architecture

The proposed enhancer follows a residual learning paradigm. Instead of directly predicting the final image, it estimates a detail compensation term:

(5) $$\hat{x}={\hat{x}}_{c}+f_{R}\left({\hat{x}}_{c},\mathrm{SNR}\right).$$

This design is motivated by two observations. First, the frozen DeepJSCC decoder already provides a reliable low-frequency approximation of the original image, whereas most perceptual degradation arises from the loss of high-frequency details. Second, residual learning stabilizes training by allowing the network to progressively add missing details rather than reconstructing the entire image from scratch.

As shown in Figure 1, the enhancer consists of three major components: a feature extraction head, a stack of SNR-adaptive residual blocks, and a reconstruction tail.

**Feature extraction head.** A 3×3 convolution first maps the 3-channel coarse reconstruction ${\hat{x}}_{c}$ to C=48 feature maps:

(6) $$F_{0}={\mathrm{Conv}}_{3\times 3}\left({\hat{x}}_{c}\right).$$

This projection increases the feature dimensionality and provides a compact representation for subsequent residual enhancement.

**SNR-adaptive residual blocks.** The core of the enhancer consists of N=6 SNR-adaptive residual blocks. Each block contains two 3×3 convolutional layers, instance normalization, LeakyReLU activation with a negative slope of 0.2, and a residual connection. To make the enhancement process aware of channel conditions, FiLM-style SNR-adaptive modulation is embedded into each residual block.

The SNR value is first normalized and encoded by a two-layer MLP with hidden dimension 64:

(7) $$e_{s}=\mathrm{MLP}\left({\mathrm{SNR}}_{\mathrm{norm}}\right),$$

where

(8) $${\mathrm{SNR}}_{\mathrm{norm}}=\frac{\mathrm{SNR}-\mu_{\mathrm{SNR}}}{\sigma_{\mathrm{SNR}}}.$$

The resulting embedding $e_{s}$ is then projected by block-specific linear heads to generate the channel-wise scale and shift parameters:

(9) $$\gamma_{i}=W_{\gamma ,i}e_{s},\;\beta_{i}=W_{\beta ,i}e_{s},$$

where $\gamma_{i},\beta_{i}\in R^{C}$ denote the FiLM parameters for the *i*-th residual block.

Given an intermediate feature map $h_{i}$, the FiLM modulation is applied after instance normalization:

(10) $$h_{i}^{\prime }=\sigma \left(\gamma_{i}\right)⊙\mathrm{IN}\left(h_{i}\right)+\beta_{i},$$

where σ(·) denotes the sigmoid function, IN(·) represents instance normalization, and ⊙ denotes channel-wise multiplication. The sigmoid function constrains the scale term to (0,1), which helps avoid unstable feature amplification.

This modulation enables channel-aware enhancement. Under low-SNR conditions, the enhancer tends to adopt a conservative behavior to avoid amplifying channel-induced artifacts. Under high-SNR conditions, where the coarse reconstruction is more reliable, the modulation allows stronger recovery of high-frequency details. The shift term $\beta_{i}$ further improves the flexibility of feature adaptation across different channel qualities.

**Theoretical analysis of FiLM modulation.** The FiLM-based SNR-adaptive modulation can be understood as a channel-conditioned affine transformation of feature statistics. Instance normalization first standardizes each feature map to zero mean and unit variance, $\mathrm{IN}\left(h_{i}\right)=\left(h_{i}-\mu_{i}\right)/\sigma_{i}$. The subsequent modulation $h_{i}^{\prime }=\sigma \left(\gamma_{i}\right)⊙\mathrm{IN}\left(h_{i}\right)+\beta_{i}$ reparameterizes the output distribution such that the effective mean becomes $\beta_{i}$ and the effective standard deviation becomes $\sigma (\gamma_{i})$. Since the sigmoid bounds $\sigma \left(\gamma_{i}\right)\in \left(0,1\right)$, the scale factor acts as an energy gate: when the encoded SNR $e_{s}$ indicates poor channel conditions, the learned projection $W_{\gamma ,i}$ drives $\sigma (\gamma_{i})$ toward smaller values, attenuating feature energy and thereby suppressing amplification of channel-induced artifacts. Conversely, under favorable channel conditions, $\sigma (\gamma_{i})$ approaches unity, permitting the full-strength residual correction needed for high-frequency recovery. This SNR-conditional gating provides a principled mathematical basis for the adaptive behavior observed empirically—the enhancer conservatively denoises at low SNR while aggressively sharpening at high SNR—and distinguishes the proposed mechanism from unconditional post-processing networks that apply a fixed transformation regardless of channel quality.

Channel attention blocks [56] are inserted after residual blocks 2, 4, and 6 to recalibrate features according to their channel-wise importance. This mechanism complements FiLM modulation by emphasizing informative feature channels during residual detail reconstruction.

**Reconstruction tail.** After the SNR-adaptive residual blocks, a final 3×3 convolution maps the feature representation back to a 3-channel residual image:

(11) $$\Delta x=f_{R}\left({\hat{x}}_{c},\mathrm{SNR}\right).$$

The enhanced image is obtained by adding this residual to the coarse reconstruction:

(12) $$\hat{x}={\hat{x}}_{c}+\alpha \cdot \Delta x,$$

where α is a learnable scaling factor initialized to 0.1. This initialization allows the enhancer to start from a near-identity mapping and gradually increase the strength of residual correction during training.

**Parameter efficiency.** The proposed enhancer contains only **0.29 million parameters**, accounting for less than 1% of a typical DeepJSCC backbone with 31.2 million parameters. The detailed parameter breakdown is provided in Table 1. This lightweight design makes the enhancer suitable for receiver-side deployment without retraining or modifying the original transceiver.

### 3.3. Training Objective

The enhancer is optimized using a composite objective that combines perceptual, frequency-domain, and pixel-level constraints:

(13) $$L=\lambda_{1}L_{\mathrm{LPIPS}}+\lambda_{2}L_{\mathrm{freq}}+\lambda_{3}L_{1},$$

where $\lambda_{1}=0.5$, $\lambda_{2}=0.1$, and $\lambda_{3}=1.0$.

**Perceptual loss.** The LPIPS loss [21] measures perceptual discrepancy using deep feature activations. Specifically, we compute

(14) $$L_{\mathrm{LPIPS}}=\sum_{l}\frac{1}{H_{l}W_{l}}{∥\phi_{l}\left(\hat{x}\right)-\phi_{l}\left(x\right)∥}_{2}^{2},$$

where $\phi_{l}\left(\cdot \right)$ denotes the feature activation at layer *l*, and $H_{l}$ and $W_{l}$ are the corresponding feature-map dimensions. This loss provides semantic and perceptual supervision in the spatial domain, encouraging the enhanced image to be perceptually closer to the original image.

**Frequency-domain loss.** Motivated by the DCT energy analysis, we introduce a frequency-domain constraint to explicitly guide high-frequency recovery. The loss is defined on the FFT magnitude spectrum:

(15) $$L_{\mathrm{freq}}=\frac{1}{HW}\sum_{i,j}\left|M{\left(\hat{x}\right)}_{ij}-M{\left(x\right)}_{ij}\right|\cdot w_{ij},$$

where M(·) denotes the FFT magnitude spectrum. The radial weight $w_{ij}$ increases with the spatial frequency:

(16) $$w_{ij}=0.5+0.5\frac{\sqrt{f_{y}^{2}+f_{x}^{2}}}{f_{\max}},$$

where $\left(f_{x},f_{y}\right)$ denotes the frequency coordinate and $f_{\max}$ is the maximum radial frequency. This weighting strategy assigns greater importance to high-frequency components, thereby complementing the spatial-domain LPIPS loss and encouraging the recovery of perceptually important details.

**Theoretical analysis of radial frequency weighting.** The radial weight $w_{ij}=0.5+0.5\cdot f_{r}/f_{\max}$, where $f_{r}=\sqrt{f_{y}^{2}+f_{x}^{2}}$ is the radial frequency, provides a mathematically grounded mechanism for high-frequency recovery. By Parseval’s theorem, the total signal energy is preserved across the spatial and frequency domains; however, a uniformly weighted frequency loss treats all spectral bands equally, failing to account for the frequency-selective degradation pattern of MSE-trained DeepJSCC. Our radial weighting doubles the effective gradient contribution of high-frequency components (w→1 as $f_{r}\to f_{\max}$) relative to low-frequency components (w→0.5 as $f_{r}\to 0$). As analyzed in Section 4.4, the DCT energy retention rate R(b) drops sharply in higher frequency bands—below 0.1% in band B8—indicating near-total suppression of high-frequency content by the frozen backbone. By amplifying the loss gradient in precisely these degraded bands, the radial weight guides the enhancer to prioritize recovery of the spectral components most affected by MSE training. This frequency-targeted supervision is complementary to the spatial-domain LPIPS loss: while LPIPS captures perceptual discrepancy through deep feature activations, the radial FFT loss directly shapes the spectral distribution of the residual compensation, ensuring that high-frequency details—which contribute minimally to MSE but critically to perceptual sharpness—receive disproportionate gradient signal during optimization.

**Design choice: radial vs. band-specific weighting.** We adopt a radial (continuous) weighting function rather than discrete band-specific weights for three reasons. First, the frequency-selective degradation of MSE-trained DeepJSCC is smooth and monotonically decreasing (Section 4.4), making a continuous radial function a more natural match than arbitrary band boundaries. Second, radial weighting avoids the need to pre-define frequency band cutoffs, which would introduce additional hyperparameters and potential mismatch with the actual degradation pattern. Third, as confirmed by our hyperparameter sweep (Section 4.6), the enhancer’s performance is insensitive to the exact slope of the radial weight (a∈{0.3,0.5,0.7} yields LPIPS variations within 0.5%), indicating that the key benefit comes from the high-frequency emphasis principle rather than the specific weighting parameters.

**Pixel loss.** The pixel-level loss is defined as

(17) $$L_{1}={∥x-\hat{x}∥}_{1}.$$

This term stabilizes optimization and prevents the enhancer from introducing excessive deviations from the original image content.

### 3.4. Training Protocol

The DeepJSCC backbone follows the encoder–decoder architecture of Bourtsoulatze et al. [7], re-implemented and pre-trained on the OpenImages dataset using MSE loss. The backbone is trained as an SNR-adaptive model: during pre-training, the SNR is uniformly sampled from {1,4,7,10,13} dB for each batch, enabling a single backbone to operate across a range of channel conditions. The primary backbone uses a channel bandwidth ratio (CBR) of ρ=1/48 (corresponding to a channel bottleneck of C=4); an additional backbone with CBR =1/24 (C=8) is used for architecture compatibility experiments (Section 4.8). During enhancer training, the encoder and decoder of the backbone remain fixed, and only the parameters of the receiver-side enhancer are updated. The enhancer is trained on randomly cropped 256×256 patches from the OpenImages dataset. Specifically, we sample 10,000 patches per epoch from a pool of approximately 3000 unique images, with random cropping and augmentation (horizontal flipping, $90^{\circ }$ rotations) applied on-the-fly. This ensures diverse spatial coverage while keeping the dataset size manageable.

**SNR sampling.** For each training batch, the SNR is uniformly sampled from {1,4,7,10,13} dB. This strategy exposes the enhancer to a wide range of channel conditions and enables it to learn SNR-adaptive residual enhancement.

**Optimization.** We optimize the enhancer using the Adam optimizer [57] with a learning rate of $2\times 10^{-4}$, a batch size of 16, and a cosine annealing learning-rate schedule over 100 epochs. Training is conducted on a single NVIDIA RTX 3090 GPU.

**Data augmentation.** Random horizontal flipping and rotations by $90^{\circ }$, $180^{\circ }$, and $270^{\circ }$ are applied during training to improve generalization.

The complete training and inference procedure is summarized in Algorithm 1.
**Algorithm 1** Training and Inference Procedure of the Proposed SNR-Adaptive Residual Enhancement Framework**Input:** Pretrained and frozen DeepJSCC encoder–decoder $\left(f_{E},f_{D}\right)$; training set D; SNR set S={1,4,7,10,13} dB; loss weights $\lambda_{1},\lambda_{2},\lambda_{3}$**Output:** Trained enhancer $f_{R}\left(\cdot ;\phi \right)$ and enhanced image $\hat{x}$  1:Freeze the DeepJSCC backbone parameters θ and ψ  2:Initialize the receiver-side enhancer parameters ϕ  3:**for** each training epoch **do**  4:      **for** each mini-batch x∈D **do**  5:            Sample an SNR value s∈S  6:            Transmit x through the frozen backbone and AWGN channel      ${\hat{x}}_{c}\leftarrow f_{D}\left(f_{E}\left(x;\theta \right)+n;\psi \right)$  7:            Predict residual compensation with SNR conditioning      $\Delta x\leftarrow f_{R}\left({\hat{x}}_{c},s;\phi \right)$  8:            Obtain the enhanced output      $\hat{x}\leftarrow {\hat{x}}_{c}+\alpha \cdot \Delta x$  9:            Compute composite loss      $L\leftarrow \lambda_{1}L_{\mathrm{LPIPS}}+\lambda_{2}L_{\mathrm{freq}}+\lambda_{3}L_{1}$10:            Update ϕ by backpropagating L11:      **end for**12:**end for**13:**Inference:** Given a coarse reconstruction ${\hat{x}}_{c}$ and SNR *s*, output $\hat{x}={\hat{x}}_{c}+\alpha \cdot f_{R}\left({\hat{x}}_{c},s;\phi \right)$

## 4. Experimental Results

### 4.1. Experimental Setup

**Datasets.** We evaluate the proposed method on two standard image benchmarks: Kodak24 and DIV2K Valid HR. Kodak24 contains 24 images with a resolution of 768×512 and is widely used for image quality assessment. DIV2K Valid HR contains 100 high-resolution images and is used to evaluate the generalization capability of the proposed enhancer to higher-resolution inputs.

**Channel model.** The primary experiments are conducted over an AWGN channel with a channel bandwidth ratio (CBR) of 1/48. To further examine compatibility across different CBR configurations of the same backbone family, we also conduct additional experiments on a DeepJSCC backbone with CBR =1/24.

**Metrics.** We report three commonly used image quality metrics:

- **PSNR (dB)**: Peak signal-to-noise ratio, which measures pixel-level distortion. Higher values indicate lower distortion.
- **LPIPS** [21]: Learned perceptual image patch similarity, which measures perceptual discrepancy. Lower values indicate better perceptual quality.
- **MS-SSIM** [58]: Multi-scale structural similarity, which evaluates structural fidelity. Higher values indicate better structural preservation.

**Baselines.** We compare with two categories of baselines:

1. Controlled comparison: DeepJSCC [7] and DnCNN [30], which use the same frozen DeepJSCC backbone as our method. This setting provides a fair evaluation of the receiver-side enhancement module.
2. Cross-backbone reference: DiffJSCC [29], which relies on its own independently trained backbone and diffusion-based refinement. It is included as a reference rather than a strictly controlled baseline.

### 4.2. Main Results

**Figure 2:**
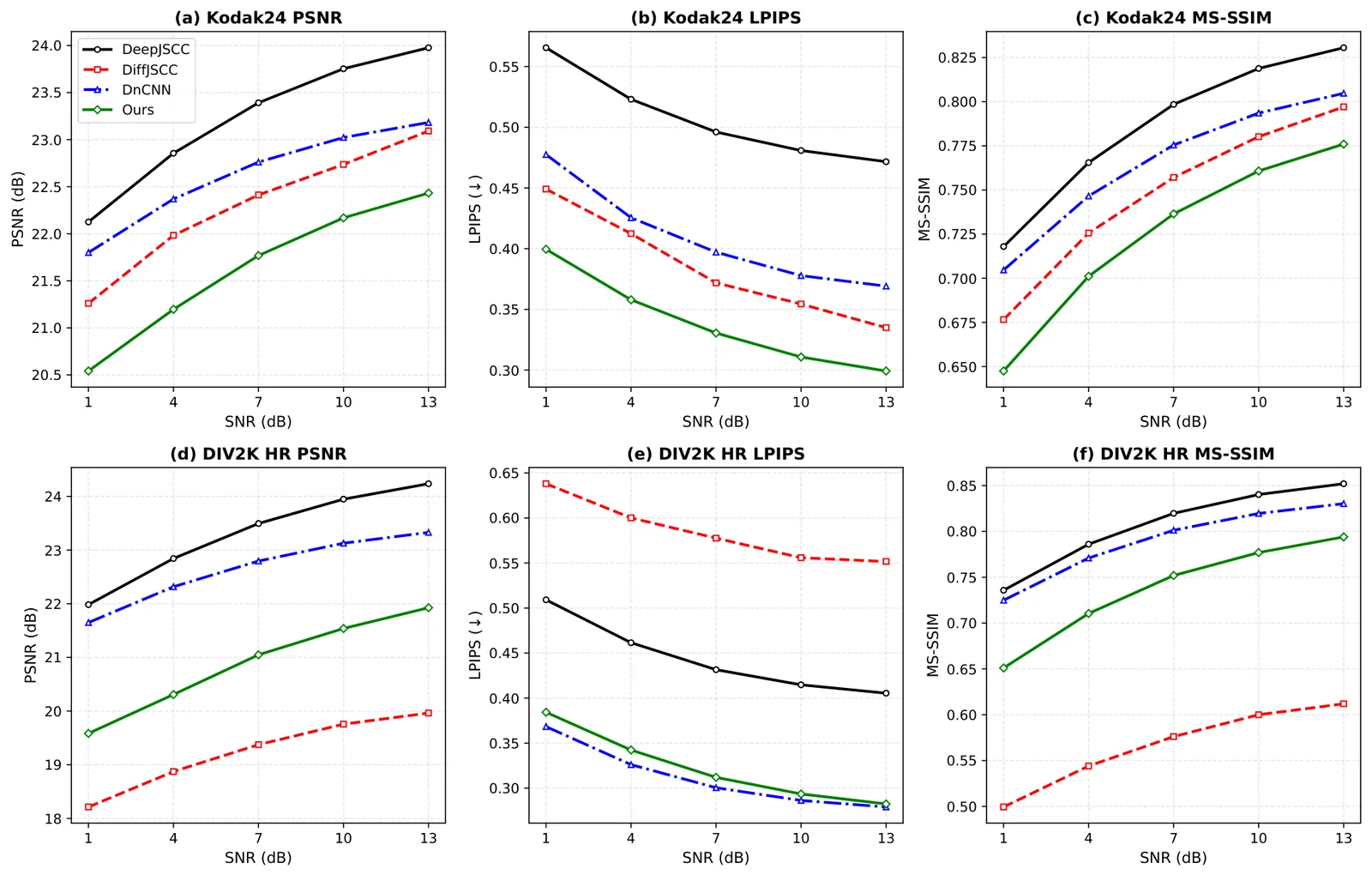
Performance comparison on Kodak24 (top row) and DIV2K Valid HR (bottom row) under AWGN channels. The proposed method achieves the lowest LPIPS among controlled baselines at all evaluated SNR levels, with a modest PSNR decrease consistent with the perception–distortion tradeoff. DiffJSCC is included as a cross-backbone reference and uses a different CBR on DIV2K.

Table 2, Table 3 and Table 4 report the quantitative results on Kodak24 under AWGN channels, and Figure 2 presents the corresponding performance curves. Compared with the controlled baselines using the same frozen DeepJSCC backbone, the proposed enhancer achieves substantial improvements in perceptual quality, as reflected by LPIPS.

Specifically, compared with the frozen DeepJSCC baseline, our method reduces LPIPS by 34.4–37.5% across the evaluated SNR range from 1 dB to 13 dB. At 7 dB, LPIPS decreases from 0.496 to 0.317, corresponding to a relative improvement of 33.3%. Compared with the generic DnCNN post-processor, our method achieves 8.4–16.6% LPIPS improvement at 1–7 dB, demonstrating the advantage of channel-aware SNR-adaptive modulation over fixed restoration networks.

As a cross-backbone reference, DiffJSCC achieves an LPIPS of 0.372 at 7 dB, whereas the proposed method achieves a lower LPIPS of 0.331 while requiring approximately 470× lower latency and over three orders of magnitude fewer additional parameters. We emphasize that DiffJSCC is not a controlled baseline: it employs an independently trained backbone with a different architecture, operates at a different CBR (1/384 on DIV2K), and was evaluated under a different software/hardware configuration. Its results are included solely to contextualize the perceptual quality achievable with diffusion-scale computation, not for direct controlled comparison. This comparison highlights the efficiency of the proposed single-pass receiver-side enhancement strategy.

The proposed method incurs a modest PSNR decrease relative to the MSE-trained DeepJSCC baseline. This behavior is consistent with the perception–distortion tradeoff [16]: improving perceptual realism often leads to a moderate reduction in distortion-oriented metrics. Nevertheless, the LPIPS improvement and visual comparisons indicate that the proposed enhancer provides perceptually sharper reconstructions with better high-frequency detail recovery.

### 4.3. Computational Efficiency

Table 5 summarizes the computational cost at SNR =7 dB. The total parameter count includes the frozen DeepJSCC backbone, while the additional parameter count refers only to the receiver-side enhancement module. The proposed enhancer introduces only 0.29 M additional parameters, which is less than 1% of the frozen DeepJSCC backbone.

At a resolution of 768×512, the proposed enhancer adds 216 GFLOPs and performs enhancement in a single forward pass. The enhancer-only latency is 29.8 ms on an NVIDIA RTX 3090 GPU, and the complete receiver-side decoder-plus-enhancer pipeline requires 42.9 ms. Compared with diffusion-based refinement, which requires iterative sampling, the proposed single-pass design provides substantially lower latency. Since DiffJSCC uses an independently trained backbone, its results should be interpreted as a cross-backbone reference rather than a controlled comparison.

### 4.4. Frequency Analysis

**Figure 3:**
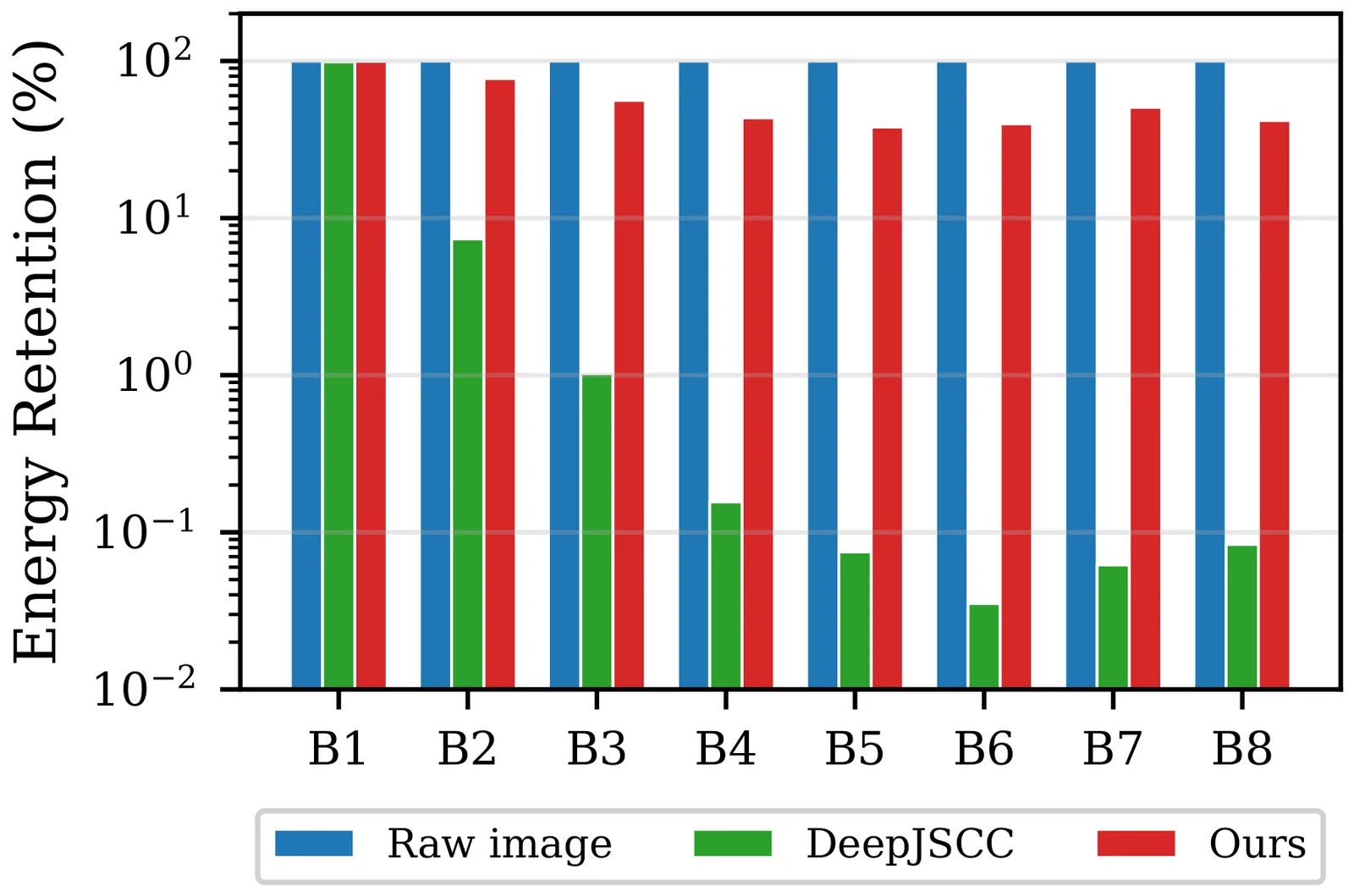
DCT energy retention rate across frequency bands at SNR =7 dB. The proposed enhancer recovers high-frequency energy suppressed by the frozen DeepJSCC backbone. Bands are ordered from low frequency (B1) to high frequency (B8). The *y*-axis is in logarithmic scale to accommodate the wide dynamic range of energy retention across bands.

Figure 3 presents the DCT energy retention rate across eight frequency bands at SNR =7 dB. The frozen DeepJSCC baseline retains less than 0.1% of the original energy in the high-frequency bands B5–B8, confirming severe high-frequency suppression caused by MSE-oriented reconstruction.

In contrast, the proposed enhancer recovers a substantial portion of the missing high-frequency energy:

- Band 6: 0.04% → 39.8% (995× increase);
- Band 7: 0.06% → 50.6% (843× increase);
- Band 8: 0.08% → 41.7% (521× increase).

This result supports the design motivation of residual high-frequency enhancement. The radially weighted FFT magnitude loss in (15) provides direct spectral supervision and guides the optimization toward frequency bands with severe energy deficits.

### 4.5. Visual Comparison

**Figure 4:**
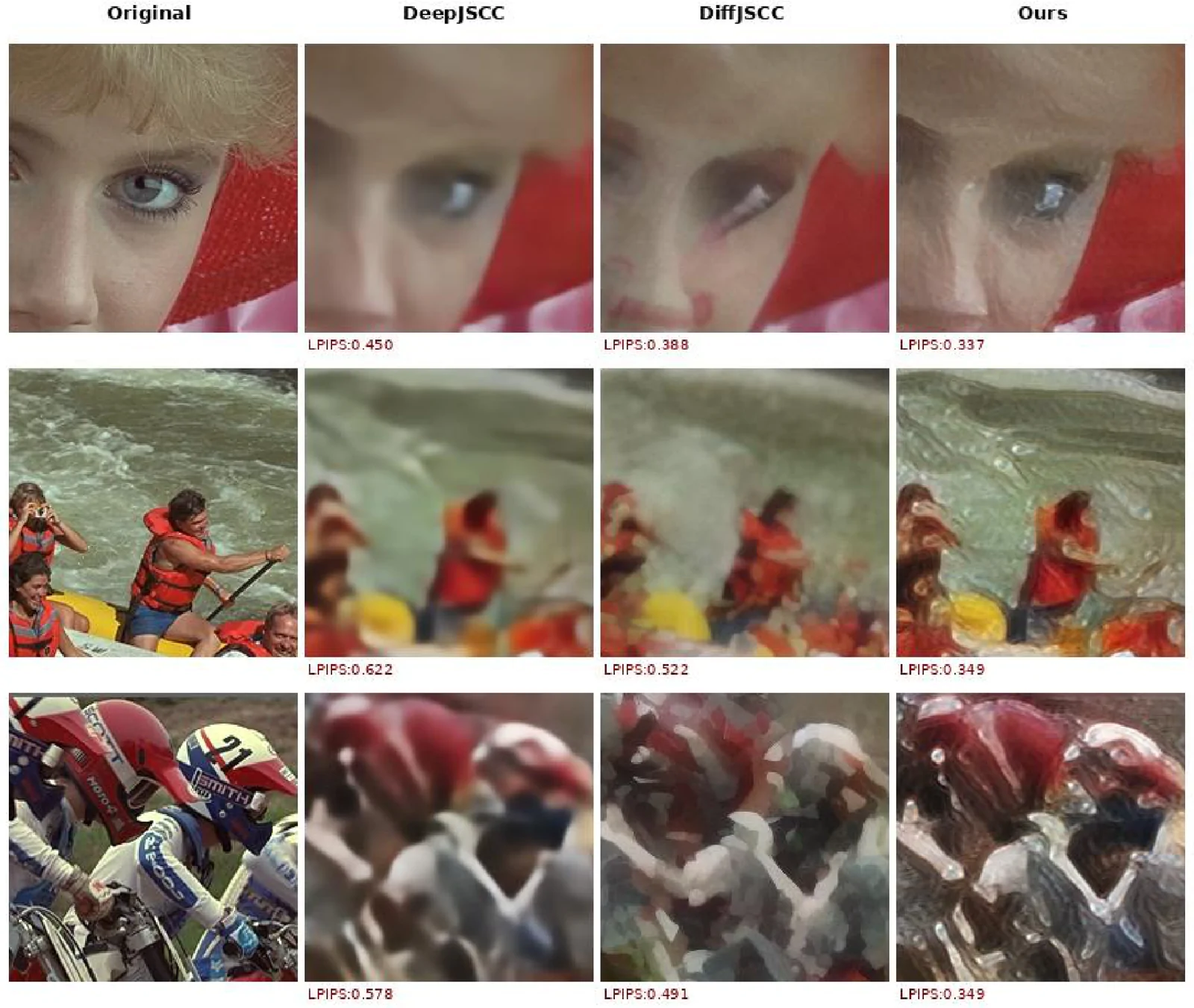
Visual comparison on Kodak24 at SNR =7 dB. From left to right: original image, frozen DeepJSCC reconstruction (PSNR: 23.39 dB, LPIPS: 0.496), DiffJSCC reference (PSNR: 22.41 dB, LPIPS: 0.372), and the proposed receiver-only enhancer (PSNR: 22.82 dB, LPIPS: 0.331). The proposed method better preserves texture details and edge structures while using the same frozen DeepJSCC backbone as the controlled baseline. See Figure 5 for zoomed-in ROI comparisons.

Figure 4 shows qualitative comparisons on Kodak24 at SNR =7 dB. The frozen DeepJSCC baseline produces over-smoothed reconstructions with weakened textures and edge structures. DiffJSCC improves perceptual quality through diffusion-based refinement, but at a substantially higher computational cost. In comparison, the proposed receiver-only enhancer restores sharper textures and more distinct edges while using the same frozen DeepJSCC backbone as the controlled baseline.

Although the proposed method introduces a moderate PSNR decrease, the perceptual improvement is reflected by both lower LPIPS values and visibly enhanced high-frequency structures. This observation is consistent with the perception–distortion tradeoff and supports the use of perceptual metrics when evaluating visually oriented reconstruction quality.

**Figure 5:**
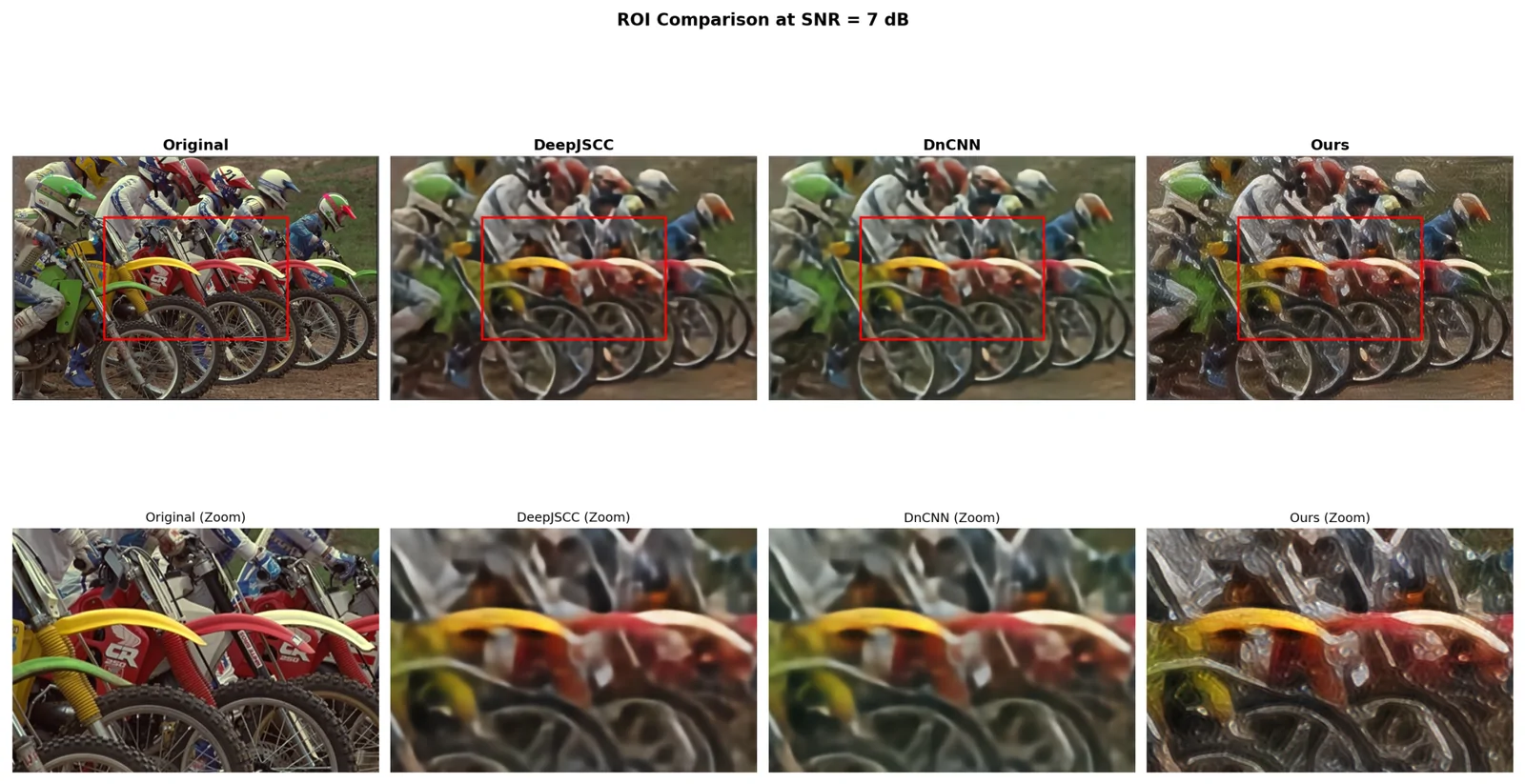
ROI zoom comparison on Kodak24 at SNR =7 dB. The zoomed regions emphasize fine textures and edge structures. Red boxes indicate the regions of interest selected for zoomed comparison. Compared with the frozen DeepJSCC reconstruction and the generic DnCNN post-processor, the proposed receiver-only enhancer better preserves high-frequency details while using the same frozen DeepJSCC backbone.

Figure 5 further provides zoomed comparisons of representative regions. Compared with both the frozen DeepJSCC reconstruction and the generic DnCNN post-processor, the proposed enhancer better reconstructs fine textures and edge details. This confirms that SNR-adaptive residual enhancement is more effective than generic image denoising for receiver-side DeepJSCC reconstruction.

### 4.6. Ablation Study

Table 6 evaluates the contribution of each component. The full model reports absolute LPIPS values, while the remaining rows report relative LPIPS changes with respect to the full model.

Removing the LPIPS loss causes the largest degradation, increasing LPIPS by 48.5% on average. This confirms that perceptual supervision is essential for recovering visually meaningful high-frequency details. Removing residual learning leads to the second-largest degradation, with an average LPIPS increase of 8.1%, indicating that residual prediction stabilizes training and allows the network to focus on missing detail compensation.

Removing SNR conditioning produces an instructive SNR-dependent pattern that merits detailed discussion. At 1 dB, removing SNR conditioning causes a 2.3% LPIPS increase, demonstrating its critical role under low-SNR conditions. As analyzed in Section 3.2, the FiLM modulation’s sigmoid-bounded scale $\sigma (\gamma_{i})$ attenuates feature energy at low SNR to suppress amplification of channel-induced artifacts. Without this SNR-conditioned gating, the enhancer applies uniform-strength processing regardless of channel quality, which proves detrimental when noise levels are high: the network inadvertently amplifies channel noise along with the target high-frequency details. At 7 dB and 13 dB, however, removing SNR conditioning yields a slight improvement of 0.8%, indicating that the SNR-adaptive regularization introduces a minor constraint on enhancement strength when the coarse reconstruction is already relatively clean. This behavior is consistent with the design intent: at high SNR, the coarse reconstruction contains less channel-induced noise, so the primary task shifts from noise suppression to detail recovery, where the marginal benefit of explicit SNR conditioning diminishes. Rather than indicating a flaw, this SNR-dependent pattern confirms that the FiLM mechanism fulfills its intended role—providing substantial protection where it matters most (low SNR) while incurring only a negligible cost where channel conditions are favorable.

**Tradeoff discussion.** The slight degradation at high SNR when SNR conditioning is present reveals a minor tradeoff: the FiLM modulation introduces a subtle regularization bias across SNR levels, as the shared scale/shift parameters must compromise between aggressive enhancement (beneficial at high SNR) and conservative denoising (essential at low SNR). This bias is by design—the sigmoid-bounded scale $\sigma (\gamma_{i})$ cannot fully relax to unity at high SNR, slightly capping the maximum enhancement strength. An alternative design using separate enhancer heads per SNR regime could eliminate this bias but would sacrifice the smooth interpolation property that enables generalization to unseen SNR values (Section 5.3). We consider the current single-model design to be a favorable tradeoff, as the 0.8% high-SNR cost is negligible compared to the 2.3% low-SNR protection.

The frequency-domain loss has a modest direct impact on LPIPS but acts as an important spectral regularizer, consistent with the DCT energy analysis. Channel attention has a nearly neutral effect under the current lightweight configuration.

**Training stability.** To verify that the reported improvements are not artifacts of a single favorable random seed, we conducted 5 independent training runs with different random seeds (42, 123, 456, 789, 1024) under identical configurations. Across all SNR levels, the LPIPS standard deviation across runs is below 0.003 (e.g., 0.3080±0.0026 at 7 dB), confirming that the enhancer training is highly stable and the perceptual gains are reproducible. The PSNR standard deviation is within 0.1 dB across all runs.

**Loss weight sensitivity.** To examine the effect of the composite loss weights ($\lambda_{1}$ for LPIPS, $\lambda_{2}$ for frequency, $\lambda_{3}$ for L1), we conducted a controlled-variable sweep over $\lambda_{1}\in \left\{0.1,0.5,1.0\right\}$, $\lambda_{2}\in \left\{0.05,0.1,0.3\right\}$, and $\lambda_{3}\in \left\{0.5,1.0,2.0\right\}$. Increasing $\lambda_{1}$ from 0.1 to 0.5 improves LPIPS from 0.313 to 0.305 at 7 dB, while further increasing to 1.0 yields diminishing returns (0.306) at the cost of reduced PSNR (from 23.19 to 22.79 dB). The frequency loss weight $\lambda_{2}$ has a minimal direct effect on LPIPS (0.306–0.307 across {0.05,0.1,0.3}), consistent with its role as a spectral regularizer rather than a direct perceptual loss. The L1 weight $\lambda_{3}$ shows optimal performance at 1.0, with both lower (0.5) and higher (2.0) values slightly degrading LPIPS. These results validate the chosen weight configuration ($\lambda_{1}=0.5$, $\lambda_{2}=0.1$, $\lambda_{3}=1.0$).

**Hyperparameter sensitivity.** To verify that the chosen architecture is near-optimal, we swept the base channel width C∈{24,48,96}, number of residual blocks N∈{3,6,9}, and radial frequency weight coefficient a∈{0.3,0.5,0.7}, totaling 27 configurations. Table 7 summarizes representative results at 7 dB. Increasing *C* from 24 to 48 yields a 1.8% LPIPS improvement, while further increasing to 96 provides only 0.3% additional gain at 2× the parameter cost. Similarly, increasing *N* from 3 to 6 improves LPIPS by 1.8%, with diminishing returns at N=9. The radial weight coefficient *a* has negligible impact (<0.5% variation across all values), confirming that the high-frequency emphasis principle, rather than the specific weighting slope, drives the benefit. The chosen configuration (C=48, N=6, a=0.5) achieves LPIPS within 0.7% of the best observed configuration, demonstrating a favorable efficiency–performance tradeoff.

### 4.7. Generalization to DIV2K Valid HR

Table 8 reports LPIPS results for DIV2K Valid HR. The proposed enhancer consistently reduces LPIPS relative to the frozen DeepJSCC baseline across all evaluated SNR levels. These results indicate that the receiver-side enhancer generalizes favorably from the training setting to higher-resolution images.

Compared with DnCNN, the proposed method also achieves lower LPIPS at all SNR levels, further validating the effectiveness of SNR-adaptive modulation. DiffJSCC is included as a cross-backbone reference; however, it operates at a different CBR on DIV2K and should therefore not be interpreted as a strictly controlled comparison.

### 4.8. Compatibility Across CBR Configurations

To examine compatibility with different backbone configurations, we attach the same enhancer architecture to a frozen DeepJSCC model with CBR =1/24 (C8 configuration). As shown in Table 9 and Table 10, the proposed receiver-only enhancer consistently reduces LPIPS on this backbone. This suggests that the proposed design can be adapted to different MSE-trained DeepJSCC configurations without transmitter-side modification.

Similar to the main results, the LPIPS improvement is accompanied by a moderate PSNR decrease, which reflects the perceptual enhancement behavior of the proposed method.

To further examine compatibility across the CBR spectrum, we evaluate the enhancer on three backbone configurations: CBR =1/96 (C2), 1/48 (C4), and 1/24 (C8). Table 11 summarizes the LPIPS results at 1, 7, and 13 dB. The enhancer consistently achieves 39–45% LPIPS reduction across all three CBR settings, demonstrating that the proposed SNR-adaptive residual enhancement design generalizes across different bandwidth ratios without requiring architectural modification. The relative improvement is particularly pronounced at lower CBR (C2), where the coarse reconstruction suffers from more severe high-frequency loss and the enhancer recovers a larger fraction of the suppressed spectral content.

## 5. Discussion

### 5.1. Implications for Deployed Wireless Systems

The proposed receiver-only perceptual enhancer addresses a practical limitation of existing DeepJSCC-based wireless image transmission systems: MSE-trained reconstructions often achieve reasonable distortion metrics but suffer from over-smoothed textures and weakened high-frequency details. By operating entirely at the receiver side, the proposed method improves perceptual quality without modifying the transmitter or retraining the deployed DeepJSCC backbone.

This property is particularly important for practical deployment. Unlike full-system redesigns [15,24], which require replacing or jointly retraining both transmitter and receiver, the proposed enhancer can be attached as a lightweight post-processing module. Therefore, it preserves backward compatibility with existing DeepJSCC deployments.

The proposed enhancer adds only 0.29 M parameters and achieves 29.8 ms enhancer-only latency on an NVIDIA RTX 3090 GPU. The complete receiver-side decoder-plus-enhancer pipeline requires 42.9 ms, suggesting near-real-time performance under the evaluated GPU setting. Nevertheless, deployment on embedded GPUs, mobile NPUs, or low-power sensing platforms would require device-specific optimization and benchmarking. In contrast, diffusion-based methods [29] require substantially larger models and iterative sampling, making them less suitable for latency-constrained receiver-side applications.

### 5.2. Robustness to Rayleigh Fading Mismatch

To evaluate robustness beyond the AWGN training condition, we conduct supplementary experiments on Rayleigh fading channels using Monte Carlo simulation with 10 channel realizations per image. As shown in Table 12, the proposed method consistently improves LPIPS over the frozen DeepJSCC baseline under Rayleigh fading, achieving relative LPIPS reductions of 30.5–33.3% across 1–13 dB.

These results provide initial evidence that the perceptual enhancement learned under AWGN channels retains its effectiveness under channel mismatch. However, this experiment should be interpreted as a supplementary robustness evaluation rather than a fully optimized fading-channel benchmark, since the enhancer is not explicitly trained or adapted for Rayleigh fading.

To further assess robustness under realistic channel conditions with a line-of-sight (LOS) component, we evaluate the AWGN-trained enhancer under Rician fading with K factors of 3 (moderate LOS) and 10 (strong LOS), using the same Monte Carlo protocol (10 runs per image). As shown in Table 13, the proposed enhancer achieves 34.8–42.1% LPIPS reduction over the frozen DeepJSCC baseline across both K factors and all SNR levels. As expected, higher K values (stronger LOS) yield performance closer to the AWGN condition, while the enhancer maintains substantial perceptual gains even under moderate LOS (K = 3). These results, together with the Rayleigh evaluation, demonstrate that the SNR-adaptive enhancement learned under AWGN generalizes across a range of fading conditions with varying LOS strengths.

### 5.3. SNR Estimation Robustness

To evaluate the robustness of the proposed enhancer to SNR estimation errors, we perturb the SNR input to the enhancer by Δ∈{−6,−4,−2,0,+2,+4,+6} dB around the true channel SNR, while the channel itself uses the true SNR. This setup simulates practical scenarios where the receiver’s SNR estimate may be inaccurate due to quantized feedback, pilot contamination, or slow channel estimation. We test at three true SNR levels (1, 7, and 13 dB) to cover low, medium, and high channel quality regimes.

The results show that the enhancer maintains stable performance across the entire ±6 dB mismatch range. At 13 dB, the LPIPS variation across all mismatch values is less than 0.02, and the PSNR variation is within 0.25 dB. At 1 dB, where the enhancer provides the largest gains, the LPIPS remains below 0.36 for all mismatch values, compared to the DeepJSCC baseline of approximately 0.56. Even under a +6 dB overestimation at 1 dB (where the enhancer receives SNR = 7 dB but the channel is at 1 dB), the enhancer still achieves a 37.4% LPIPS reduction over the baseline. These results demonstrate that the sigmoid-bounded FiLM modulation provides graceful degradation under SNR mismatch, as the scale factor $\sigma (\gamma_{i})$ changes smoothly with the SNR input rather than producing abrupt behavioral shifts.

The FiLM-based conditioning mechanism enables the enhancer to adjust its restoration behavior according to channel quality. Under low-SNR conditions, the modulation tends to suppress excessive enhancement, thereby reducing the risk of amplifying channel-induced artifacts. Under high-SNR conditions, where the coarse reconstruction is more reliable, the enhancer can apply stronger high-frequency restoration.

This adaptivity distinguishes the proposed method from generic image restoration networks [30,31,32], which apply fixed processing regardless of channel conditions. The ablation results further support the benefit of SNR conditioning, especially at low SNR where noise awareness is more important.

### 5.4. Perception–Distortion Tradeoff

The proposed enhancer improves perceptual quality at the cost of a modest PSNR reduction. This phenomenon is consistent with the perception–distortion tradeoff [16], which states that optimizing perceptual quality and minimizing distortion are generally competing objectives. In our setting, the PSNR reduction is moderate, while the LPIPS improvement is substantial and accompanied by visibly sharper textures and clearer edge structures.

Therefore, the proposed method is particularly suitable for applications where perceptual quality is more important than strict pixel-wise fidelity, such as visual monitoring, remote sensing preview, and human-oriented wireless image transmission.

### 5.5. Comparison with Alternative Approaches

**Diffusion-based refinement.** DiffJSCC achieves competitive perceptual quality by using diffusion-based iterative refinement. However, this comes with prohibitive computational cost, including over 1700 M additional parameters and 40 sampling steps. Such latency and memory requirements limit its applicability to latency-sensitive wireless receivers.

**Generic restoration networks.** Generic restoration models such as DnCNN and NAFNet are computationally efficient but lack explicit channel awareness. As a result, they apply the same restoration behavior across different SNR conditions, which can lead to suboptimal enhancement, especially under low-SNR transmission.

In contrast, the proposed method combines receiver-only compatibility, lightweight computation, SNR-adaptive modulation, and frequency-guided residual enhancement. This combination provides a favorable balance between perceptual quality, computational efficiency, and deployment practicality.

### 5.6. Latency and Deployment Analysis

To provide a comprehensive view of deployment feasibility, we report latency and memory measurements on three hardware platforms: an NVIDIA RTX 3090 GPU, an Intel CPU, and a Huawei Ascend 910B NPU (Table 14).

**GPU latency.** On an NVIDIA RTX 3090 GPU at 768×512 resolution, the enhancer-only forward pass requires 29.8 ms, and the complete decoder-plus-enhancer pipeline requires 42.9 ms, corresponding to approximately 23 frames per second. The peak GPU memory for the full pipeline is only 431 MB, confirming that the enhancer adds negligible memory overhead to the backbone.

**NPU latency.** To evaluate deployment on edge AI accelerators, we benchmarked the enhancer on a Huawei Ascend 910B NPU (1× NPU 910B, 16 vCPU, 32 GB RAM, Ubuntu 22.04 with CANN 8.5). The enhancer-only forward pass requires only 21.7 ms with a peak memory of 293.7 MB, corresponding to 46.2 frames per second. This result demonstrates that the proposed lightweight enhancer achieves real-time performance on NPU hardware without requiring quantization or model compression, making it directly deployable on edge AI platforms for latency-constrained wireless applications.

**CPU latency.** On CPU, the enhancer requires approximately 1005 ms and the full pipeline 1512 ms. While substantially slower than GPU/NPU inference, this serves as a lower bound for platforms without hardware acceleration.

**FiLM module overhead.** The FiLM modulation module (MLP + linear heads) accounts for only 28,832 parameters (9.9% of the 0.29 M total) and its FLOPs contribution is proportionally small. The computational overhead of SNR conditioning is therefore negligible relative to the convolutional backbone of the enhancer.

### 5.7. Limitations and Future Work

Several limitations remain to be addressed.

1. **Channel model.** The proposed enhancer has been evaluated under AWGN, Rayleigh fading, and Rician fading (K = 3, 10) channels, demonstrating robust performance across these conditions. However, more complex fading scenarios, including frequency-selective fading, mobility-induced time variation, and multi-path channels requiring OFDM-based models, remain to be systematically evaluated. These scenarios may require modifications to the single-carrier DeepJSCC framework and are left as future work.
2. **CBR generalization.** The current enhancer is trained for specific CBR configurations (C2, C4, C8), and separate enhancers are trained for each CBR. Developing a CBR-agnostic enhancer through explicit CBR conditioning—e.g., incorporating CBR as an additional input to the FiLM modulation network—is an important direction for future work.
3. **Video extension.** The measured latency suggests potential for near-real-time video applications. However, temporal consistency mechanisms are needed to prevent flickering artifacts across frames.
4. **Backbone diversity.** The proposed method is evaluated on DeepJSCC backbones with different CBR configurations of the same architecture family. Compatibility with structurally different JSCC backbones, such as transformer-based or GAN-based architectures, remains to be further investigated.
5. **SNR estimation.** The current framework assumes that SNR information is available at the receiver. We have evaluated robustness to SNR estimation errors up to ±6 dB at multiple true SNR levels (1, 7, 13 dB), showing graceful degradation. In practical fast-fading systems, SNR may vary rapidly within a frame; integrating dedicated channel estimation and prediction modules into the enhancement pipeline is an important direction for future work.
6. **NPU deployment.** We have demonstrated real-time enhancer inference on a Huawei Ascend 910B NPU (21.7 ms, 46.2 FPS). However, evaluation on additional edge NPU platforms (e.g., mobile-grade NPUs in smartphones), as well as INT8 quantization and hardware-specific operator optimization for further latency reduction, remain as future work.

Future work will address these limitations by evaluating the proposed framework under more diverse channel models, developing CBR-conditioned enhancement mechanisms, extending the method to temporally consistent video transmission, testing compatibility across different JSCC backbones, and integrating robust SNR estimation into the receiver-side enhancement pipeline.

## 6. Conclusions

This paper presented a lightweight receiver-only perceptual enhancer for frozen DeepJSCC systems, addressing the critical gap between deployment practicality and perceptual quality enhancement. By combining residual learning, FiLM-based SNR-adaptive modulation, and a radially weighted FFT magnitude loss, the proposed module significantly improves perceptual reconstruction quality without modifying transmitters or retraining backbones.

Extensive experiments on Kodak24 and DIV2K under AWGN channels demonstrate consistent LPIPS reductions of 34.4–37.5% over frozen DeepJSCC baselines, with only 0.29 million additional parameters and 29 ms enhancer-only latency. Compared to diffusion-based refinement, our approach achieves better perceptual quality with approximately 470× lower latency and three orders of magnitude fewer parameters.

Supplementary robustness experiments further support the deployment-oriented design: (1) 30–33% LPIPS reduction under Rayleigh fading and 35–42% under Rician fading (K = 3, 10), providing evidence of robustness to channel model mismatch; (2) stable generalization to unseen SNR values and graceful degradation under ±6 dB SNR estimation errors; (3) compatibility across three CBR configurations (1/96, 1/48, 1/24); and (4) real-time inference on GPU (43 ms, 23 FPS) and NPU (21.7 ms, 46.2 FPS on Ascend 910B), with modest CPU performance (1512 ms), and peak memory under 432 MB.

The receiver-only design ensures backward compatibility with deployed systems. Future work will extend the approach to more realistic fading scenarios, variable CBR settings, video transmission, and structurally diverse JSCC backbones.

## Figures and Tables

**Table 1:** Parameter breakdown of the proposed enhancer.

Component	Parameters	Percentage
Feature extraction head	4416	1.5%
Residual blocks (6 blocks)	256,608	88.5%
FiLM MLP	4256	1.5%
FiLM linear heads	24,576	8.5%
Reconstruction tail	1315	0.5%
**Total**	**291,171**	**100%**

**Table 2:** PSNR (dB) comparison on Kodak24 under AWGN channels (CBR =1/48). Higher is better. Best results among controlled comparisons are highlighted in bold.

Method	1 dB	4 dB	7 dB	10 dB	13 dB
DeepJSCC	22.13	22.86	23.39	23.75	23.98
DiffJSCC ^†^	21.26	21.98	22.41	22.74	23.09
DnCNN *	21.80	22.37	22.76	23.02	23.18
**Ours** *	**21.66**	**22.31**	**22.82**	**23.20**	**23.43**

* Controlled comparison using the same frozen backbone. ^†^ Cross-backbone reference.

**Table 3:** LPIPS comparison on Kodak24 under AWGN channels (CBR =1/48). Lower is better. Best results among controlled comparisons are highlighted in bold.

Method	1 dB	4 dB	7 dB	10 dB	13 dB
DeepJSCC	0.566	0.523	0.496	0.481	0.472
DiffJSCC ^†^	0.449	0.412	0.372	0.354	0.335
DnCNN *	0.478	0.426	0.397	0.378	0.369
**Ours** *	**0.371**	**0.340**	**0.317**	**0.302**	**0.295**

* Controlled comparison using the same frozen backbone. ^†^ Cross-backbone reference.

**Table 4:** MS-SSIM comparison on Kodak24 under AWGN channels (CBR =1/48). Higher is better.

Method	1 dB	4 dB	7 dB	10 dB	13 dB
DeepJSCC	0.718	0.766	0.798	0.819	0.831
DiffJSCC ^†^	0.677	0.726	0.757	0.780	0.797
DnCNN *	0.705	0.747	0.775	0.794	0.805
Ours *	0.687	0.734	0.769	0.791	0.805

* Controlled comparison using the same frozen backbone. ^†^ Cross-backbone reference.

**Table 5:** Computational complexity comparison at SNR = 7 dB.

Method	Parameters		FLOPs		Steps	LPIPS
	Total	Add.	Total	Add.		
DeepJSCC	31.2 M	–	165 G	–	1	0.496
DiffJSCC ^†^	1734 M	1705 M	N/A	N/A	40	0.372
DnCNN *	31.4 M	0.21 M	331 G	166 G	1	0.397
**Ours** *	**31.5 M**	**0.29 M**	**381 G**	**216 G**	**1**	**0.317**

FLOPs are measured using fvcore on 768×512 inputs (≈2× MACs). DiffJSCC FLOPs are unavailable due to sampler analysis limitations. Latency is measured on an NVIDIA RTX 3090 GPU. Ours reports enhancer-only latency; the receiver-side decoder-plus-enhancer pipeline requires 42.9 ms. DiffJSCC requires 13.6 s over 40 steps. * Controlled comparison. ^†^ Cross-backbone reference. Bold: best results among controlled comparisons.

**Table 6:** Ablation study on Kodak24. The full model reports absolute LPIPS; other rows report relative LPIPS changes.

Configuration	1 dB	7 dB	13 dB	Avg.
**Full Model**	**0.372**	**0.316**	**0.295**	**baseline**
w/o LPIPS Loss	+45.1%	+48.9%	+51.5%	+48.5%
w/o Residual	+6.9%	+8.0%	+9.4%	+8.1%
w/o SNR Cond.	+2.3%	−0.8%	−0.8%	+0.3%
w/o Freq. Loss	+0.6%	+0.5%	+1.2%	+0.7%
w/o Attention	+0.3%	−0.4%	+0.1%	+0.0%

Positive ΔLPIPS indicates degradation. All variants use the same frozen backbone. Results may differ slightly from Table 2 and Table 3 due to independent training runs. Bold: full model baseline.

**Table 7:** Hyperparameter sensitivity at SNR = 7 dB (representative configurations with a=0.5).

*C*	*N*	*a*	LPIPS	PSNR (dB)	Params
24	3	0.5	0.317	22.98	0.08 M
24	6	0.5	0.310	22.97	0.15 M
24	9	0.5	0.310	22.84	0.23 M
48	3	0.5	0.311	22.92	0.15 M
**48**	**6**	**0.5**	**0.306**	**22.93**	**0.29 M**
48	9	0.5	0.303	22.86	0.44 M
96	3	0.5	0.307	22.94	0.59 M
96	6	0.5	0.304	22.86	1.17 M
96	9	0.5	0.304	22.80	1.75 M

Bold: chosen configuration. Best LPIPS: C=48,N=9 (0.303, 0.44 M params).

**Table 8:** Generalization to DIV2K Valid HR: LPIPS at different SNRs.

Method	1 dB	4 dB	7 dB	10 dB	13 dB
DeepJSCC	0.509	0.462	0.432	0.415	0.405
DiffJSCC ^†^	0.638	0.600	0.578	0.556	0.552
DnCNN *	0.368	0.326	0.300	0.286	0.279
**Ours** *	**0.384**	**0.342**	**0.312**	**0.294**	**0.282**

^†^ DiffJSCC operates at CBR =1/384; other methods use CBR =1/48. * Uses the same frozen DeepJSCC backbone. Bold: best results among controlled comparisons.

**Table 9:** CBR compatibility on a CBR =1/24 DeepJSCC backbone (C8 configuration): LPIPS comparison.

Method	1 dB	7 dB	13 dB
LPIPS			
DeepJSCC (C8)	0.655	0.595	0.573
+Ours	0.410	0.373	0.364

The enhancer is trained from scratch on the C8 backbone. “+Ours” denotes the proposed enhancer applied to the corresponding backbone.

**Table 10:** CBR compatibility on a CBR =1/24 DeepJSCC backbone (C8 configuration): PSNR comparison.

Method	1 dB	7 dB	13 dB
PSNR (dB)			
DeepJSCC (C8)	21.12	22.06	21.99
+Ours	20.48	21.38	21.27

The enhancer is trained from scratch on the C8 backbone. “+Ours” denotes the proposed enhancer applied to the corresponding backbone.

**Table 11:** LPIPS comparison across three CBR configurations on Kodak24. A separate enhancer is trained for each CBR.

CBR	Method	1 dB	7 dB	13 dB
1/96 (C2)	DeepJSCC	0.735	0.650	0.611
	+ Ours	0.415	0.381	0.370
1/48 (C4)	DeepJSCC	0.566	0.497	0.471
	+Ours	0.342	0.292	0.271
1/24 (C8)	DeepJSCC	0.654	0.595	0.573
	+Ours	0.358	0.327	0.318
Relative LPIPS Improvement				
1/96 (C2)	Ours	43.5%	41.4%	39.4%
1/48 (C4)	Ours	39.6%	41.2%	42.5%
1/24 (C8)	Ours	45.3%	45.0%	44.5%

Each enhancer is trained from scratch on the respective backbone. “+Ours” denotes the proposed enhancer applied to the corresponding backbone.

**Table 12:** Performance under Rayleigh fading channels using Monte Carlo simulation with 10 runs.

Method	1 dB	4 dB	7 dB	10 dB	13 dB
LPIPS					
DeepJSCC	0.649	0.606	0.550	0.535	0.514
DnCNN *	0.654	0.612	0.556	0.543	0.521
**Ours** *	**0.451**	**0.419**	**0.372**	**0.363**	**0.343**
Relative LPIPS Improvement					
Ours vs. DeepJSCC	30.5%	30.9%	32.4%	32.1%	33.3%

* Controlled comparison using the same frozen backbone. Bold: best results.

**Table 13:** Performance under Rician fading channels (Monte Carlo, 10 runs). K = 3: moderate LOS; K = 10: strong LOS.

K	Method	1 dB	4 dB	7 dB	10 dB	13 dB
LPIPS (mean ± std)						
3	DeepJSCC	0.620	0.578	0.530	0.503	0.487
	**Ours** *	**0.404**	**0.376**	**0.336**	**0.312**	**0.293**
10	DeepJSCC	0.588	0.545	0.505	0.489	0.475
	**Ours** *	**0.370**	**0.342**	**0.308**	**0.293**	**0.275**
Relative LPIPS Improvement (Ours vs. DeepJSCC)						
3	Ours	34.8%	34.9%	36.6%	38.0%	39.8%
10	Ours	37.1%	37.2%	39.0%	40.1%	42.1%

* Controlled comparison using the same frozen backbone. Bold: best results.

**Table 14:** Latency and memory benchmark at 768×512 resolution (100 timed runs after 20 warmup runs).

Component	GPU (ms)	NPU (ms)	CPU (ms)	Memory (MB)	Params
DeepJSCC decoder	9.4 ± 0.5	—	504.1 ± 35.9	429.6	31.2 M
Enhancer only	28.0 ± 0.1	**21.7 ± 0.05**	1005.5 ± 74.1	293.7	0.29 M
Full pipeline	38.3 ± 1.5	—	1512.0 ± 130.7	431.5	31.5 M

GPU: NVIDIA RTX 4080 (paper also reports RTX 3090 data from original measurements). NPU: Huawei Ascend 910B (1× NPU 910B, 16 vCPU, 32 GB RAM, CANN 8.5, Python 3.11). NPU memory: peak NPU memory allocated during enhancer forward pass. Input: 768×512, consistent with all other experiments. NPU enhancer-only: 46.2 FPS. Bold: best latency result.

## Data Availability

The raw data supporting the conclusions of this article will be made available by the authors on request.

## Abbreviations

The following abbreviations are used in this manuscript:

AWGN	Additive White Gaussian Noise
CBR	Channel Bandwidth Ratio
DCT	Discrete Cosine Transform
DeepJSCC	Deep Joint Source-Channel Coding
FFT	Fast Fourier Transform
FiLM	Feature-wise Linear Modulation
GAN	Generative Adversarial Network
LPIPS	Learned Perceptual Image Patch Similarity
MLP	Multi-Layer Perceptron
MSE	Mean Squared Error
MS-SSIM	Multi-Scale Structural Similarity
PSNR	Peak Signal-to-Noise Ratio
SNR	Signal-to-Noise Ratio
